# Efficacy of microwave ablation versus radiofrequency ablation for hepatocellular carcinoma: a propensity score analysis

**DOI:** 10.1007/s00261-021-03008-9

**Published:** 2021-03-06

**Authors:** Kanehiko Suwa, Toshihito Seki, Kazunori Aoi, Masao Yamashina, Miki Murata, Noriyo Yamashiki, Akiyoshi Nishio, Masaaki Shimatani, Makoto Naganuma

**Affiliations:** 1 The Third Department of Internal Medicine, Division of Gastroenterology and Hepatology, Kansai Medical University, 2-3-1 Shimmachi, Hirakata, Osaka 573-1101 Japan; 2 Kansai Medical University Medical Center, Liver Disease Center, 10-15 Fumizonocho, Moriguchi, Osaka 570-8507 Japan

**Keywords:** Hepatocellular carcinoma, Microwave ablation, Radiofrequency ablation, Local tumor progression, Recurrence-free survival

## Abstract

**Purpose:**

To evaluate the efficacy and safety of radiofrequency ablation (RFA) and new-generation microwave ablation (MWA) for the treatment of hepatocellular carcinoma (HCC).

**Methods:**

The propensity score matching method was applied to patients with HCC treated with MWA (93 patients) or RFA (156 patients) at a single institution from January 2014 to April 2020. The local tumor progression (LTP), intrahepatic distant recurrence (IDR), and recurrence-free survival (RFS) of the two matched therapies were analyzed using the Kaplan–Meier method. Cox proportional hazard models were used to identify risk factors for LTP and RFS. The therapeutic effects and complications of the two treatments were also compared.

**Results:**

The LTP, IDR, and RFS of MWA and RFA were equivalent (LTP: hazard ratio [HR] = 0.87; 95% confidence interval [95% CI] 0.36- 2.07; *P* = 0.746, IDR: HR = 1.03; 95% CI 0.61–1.73; *P* = 0.890, RFS: HR = 1.15; 95% CI 0.69–1.91; *P* = 0.566). Para-vessel lesions was the only risk factor for LTP, whereas age, previous treatment, Albumin-Bilirubin score, and tumor diameter were risk factors for RFS. On the other hand, the ablation time per nodule (6.79 ± 2.73 and 9.21 ± 4.90 min; *P* = 0.008) and number of sessions per nodule required to achieve technical success (1.16 ± 0.39 and 1.34 ± 0.57; *P* = 0.009) were significantly lower in MWA than in RFA. The major complication rate of MWA and RFA was also equivalent.

**Conclusion:**

MWA and RFA have similar therapeutic effects and safety, although MWA has advantages over RFA regarding efficacy, including shorter ablation time and fewer sessions required.

## Introduction

Hepatocellular carcinoma (HCC) is one of the leading causes of cancer-related death globally, and it is estimated that both its incidence and mortality are increasing worldwide [1]. The Barcelona Clinic Liver Cancer staging classification and treatment schedule, one of the most widely adopted liver cancer staging systems, recommends liver transplantation, resection, and ablation therapy as curative treatments for early-stage HCC [2, 3]. Thermal ablation procedures are the best option as a curative treatment for patients with HCC who cannot undergo surgical resection or liver transplantation due to their high local tumor control and minimally invasive nature [4]. Several meta-analyses suggest that thermal ablation may be favorable as a first-line treatment [5, 6]. Radiofrequency ablation (RFA) is widely used in thermal ablation, and most large-scale HCC trials have been performed using RFA [7–9]. More recently, the Emprint Ablation System™ (Covidien), a microwave ablation (MWA) system with new technology, has gained increased attention [10, 11]. This new system opens up an interesting perspective as to the optimal selection of ablation modalities for local therapy for HCC. This study adopted a propensity score matching (PSM) analysis to reduce potential confounding bias at baseline. The purpose of this study was to investigate the therapeutic efficacy and safety of MWA versus RFA systems and to analyze the predictors that might influence the superiority of one system over the other.

## Materials and methods

### Patients

This retrospective study evaluated patients treated with percutaneous thermal ablation (PTA) at the Kansai Medical University Medical Center, Liver Disease Center between January 2014 and April 2020. RFA was utilized for all patients treated before November 2017, whereas MWA was routinely used after November 2017. Exceptionally, during 2018, RF was used in three cases due to generator problems. The study group included 93 patients treated with MWA and 156 patients treated with RFA. Patients receiving palliative therapy or those with extrahepatic metastases or portal vein tumor thrombosis were excluded to assess treatment efficacy correctly. We also excluded patients with a tendency toward severe bleeding, and those whose liver function was Child–Pugh Class C with refractory ascites. All patients were treated with a curative intent and the tumors were within the Milan criteria.

The study protocol was conducted with the approval (approval no. 2018143) of the Ethics Committee of Kansai Medical University Medical Center (Moriguchi, Japan). All study procedures were performed in accordance with the ethical standards of the Clinical Research Board of Kansai Medical University Medical Center and with the 1964 Helsinki Declaration and its later amendments or comparable ethical standards. Informed consent was obtained from all participants included in the study.

### Diagnosis

Clinical diagnosis and cancer grading were performed using computed tomography (CT) during angiography (CT-A) and computed tomography during arterial portography (CT-AP). If a clinical diagnosis was difficult, the histological diagnosis of tumors was confirmed by ultrasound (US) guided fine needle biopsy.

Large vessels were defined as having a diameter greater than 3 mm, and HCC adjacent to large vessels was defined as a lesion confirmed to be in contact with large vessels on the US screen.

### Ablation procedure

Both operators who performed PTA procedures had more than 5 years of experience in intervention therapy at the start of this study and treated patients in the same manner. All ablation procedures were performed via a percutaneous approach under US guidance (TUS‑A300 Aplio300; Canon). After local anesthesia was administered as 0.5% lidocaine hydrochloride (Aspen Japan), a guide needle (MWA, 12 G × 140 mm; RFA, 14 G × 145 mm; Hakko Medical) was inserted into the vicinity of the tumor under US guidance. Subsequently, the inner needle of the guide was removed, and the antenna (electrode) was inserted through the outer needle of the guide to place the antenna (electrode) in the tumor area.

Microwave ablation was performed using an Emprint™ Ablation Generator with Thermosphere Technology with an Emprint™ Long Percutaneous Antenna (30 cm; Covidien). Radiofrequency ablation was performed using a Cool‑tip RF Generator with a Cool‑tip RF single needle (25 × 3 cm; Covidien). The output energy was optimized and adjusted appropriately according to each case. The output energy was set at 60–100 W and 80–120 W in the case of MWA and RFA, respectively. The ablation area was monitored by a real-time US, and ablations were terminated when the hyperechoic zone adequately covered the lesion on the US screen referring to the recommended time protocol for each device. If the ablation area was determined to be insufficient by the following evaluation method, additional ablation was performed at a later date.

### Evaluation of treatment effect and follow-up

Four days after ablation, the direct effect of ablation was determined by dynamic CT. Technical success was determined at each nodule and defined as a wide coagulated area around the circumference than the low-density area in the late phase of pretreatment dynamic CT. The initial technical success rate was determined after the initial ablation session, and the secondary technical success rate was determined as the proportion of successful treatment with repeated ablation.

All patients were closely followed after discharge from the hospital, and dynamic CT was performed every 3–4 months. When imaging studies revealed intrahepatic recurrence, the diagnosis was confirmed by CT angiography and/or US‑guided tumor biopsy. Local tumor progression (LTP) was defined as a recurrence of the nodule in the treatment area or the treatment area’s margin. Complications were classified according to the Society of Interventional Radiology classification of complications by outcome [12]. The follow‑up period ended in June 2020.

### Statistical analyses

In this study, PSM analysis was applied only to the cases that had technical success to focus on tumor recurrences of the two ablation systems. Propensity scores were estimated using a logistic regression model, and 1:1 patient matching was performed based on each patient’s propensity score. The variables included in the propensity score model were age, sex, background liver disease, platelet count, Albumin-Bilirubin (ALBI) score [13], α-fetoprotein (AFP), naïve or recurrence, tumor size, tumor number, adjacency to large vessels, and presence or absence of combination therapy with Conventional transcatheter arterial chemoembolization (c-TACE).

Group (MWA versus RFA) differences in clinical data were compared using Mann–Whitney U tests for continuous variables and Pearson’s Chi-square tests for categorical variables. Cumulative incidence rates for LTP, intrahepatic distant recurrence (IDR), and recurrence-free survival (RFS) were estimated using the Kaplan–Meier method, and differences between groups were compared by the log-rank test. Risk factors for LTP and RFS were assessed using Cox proportional hazard models in univariate and multivariate analyses for all patients, not just the propensity score-matched patients. Each variable in the multivariate analysis was adjusted for items that were expected to be relevant to each event. All variables with *P*-values less than 0.10 in the univariate analysis were selected for the multivariate analysis. *P*-values less than 0.05 were considered to indicate a statistically significant difference. All statistical analyses were performed using the R statistical package (R software version 3.4.1; R Foundation for Statistical Computing, Vienna, Austria).

## Results

### Patient population

The full study cohort comprised 249 patients, including 93 patients treated with MWA and 156 patients treated with RFA. The follow-up period was longer in patients treated with RFA, as RFA was routinely used at the study’s inception (median follow-up of 364 vs. 1150 days). Baseline characteristics of all patients are shown in Table 1. c-TACE using iodized oil emulsion (4–6 ml, Lipiodol, Andre Guerbet) was performed for all tumors when diagnosed as HCC with CT-A and CT-AP. Before PSM, the RFA group had significantly less para-vessel lesions than the MWA group (44.1% vs. 19.2%) but were associated with worse underlying liver disease such as lower prothrombin time, and albumin and platelet counts (86.8 vs. 77.3%, 3.9 vs. 3.7 g/dl, 14.2 vs. 11.8 × 104/µl, respectively). One hundred forty-four patients were selected during PSM, and after matching, the two groups were not significantly different in terms of baseline factors (Table 1).

**Table 1:** Baseline characteristics before and after propensity score

	Before propensity score matching			After propensity score matching		
Variables	MWA	RFA	P-value	MWA	RFA	P-value
Patient (n)	93	156		72	72	
Nodule (n)	112	185		86	86	
Observation period (days, median)	364	1150		330	1064	
Sex			0.39			0.86
Male	62	113		47	49	
Female	31	43		25	23	
Age (y)	74.17 ± 8.35	72.38 ± 9.51	0.133	74.90 ± 8.43	74.40 ± 9.19	0.734
Background			0.814			0.931
HBV	10	21		6	6	
HCV	56	93		47	44	
NBNC	27	42		19	22	
Type -naïve or recurrent			0.672			0.858
Naive	27	50		22	24	
Recurrent	66	106		50	48	
Child–Pugh class			0.627			0.661
A	76	123		58	61	
B	17	33		14	11	
PT (%)	86.86 ± 17.12	77.38 ± 13.46	< 0.001	86.56 ± 15.99	79.56 ± 14.52	0.007
ALB (g/dl)	3.96 ± 0.58	3.77 ± 0.56	0.011	3.90 ± 0.59	3.93 ± 0.54	0.79
T-Bil (mg/dl)	0.95 ± 0.53	1.02 ± 1.00	0.538	1.02 ± 0.52	0.95 ± 0.52	0.395
PLT (10^4^/µl)	14.25 ± 6.16	11.85 ± 7.30	0.008	13.64 ± 6.03	12.63 ± 6.77	0.348
ALBI score	− 2.60 ± 0.54	− 2.43 ± 0.51	0.01	− 2.53 ± 0.54	− 2.57 ± 0.50	0.628
AFP (ng/ml)	111.79 ± 613.22	119.29 ± 477.25	0.914	47.24 ± 152.64	32.28 ± 87.54	0.472
TACE			0.1			0.845
Yes	75	111		56	54	
No	18	45		16	18	
Location			< 0.001			0.862
Vessel-adjacent	41	30		25	27	
Non vessel-adjacent	52	126		47	45	
Tumor size (mm)	18.47 ± 8.29	17.52 ± 6.60	0.322	17.72 ± 6.76	17.55 ± 6.29	0.874
Tumor number	1.85 ± 1.18	1.77 ± 1.17	0.603	1.88 ± 1.17	1.88 ± 1.20	1

*MWA* Microwave ablation, *RFA* radiofrequency ablation, *HBV* hepatitis B virus, *HCV* hepatitis C virus, *NBNC* non B non-C, *PT* prothrombin time, *ALB* albumin, *T-Bil* total bilirubin, *PLT* platelet, *ALBI* albumin bilirubin grade, *AFP* α fetoprotein, *TACE* transcatheter arterial chemoembolization

### Treatment efficacy

Eighty-eight of the 93 patients in the MWA group and 140 of the 156 patients in the RFA group achieved initial technical success (94.6 vs. 89.7%), 92 patients in the MWA group and 151 patients in the RFA group achieved secondary technical success (98.9 vs. 96.7%). There was no group difference in technical success rates. In the two matched groups, the average number of sessions per nodule required to achieve technical success was significantly fewer for MWA (1.16 ± 0.39) than for RFA (1.34 ± 0.57) (*P* = 0.009). Similarly, the average ablation time per nodule was significantly shorter for MWA (6.79 ± 2.73 min) than for RFA (9.21 ± 4.90 min) (*P* = 0.008). Treated nodules whose coagulated area included 5 mm or more safety margin from the tumor were 59 out of 86 nodules for MWA and 48 out of 86 nodules for RFA, and there was no significant difference between the two groups (*P* = 0.115).

### Tumor recurrence

**Fig. 1:**
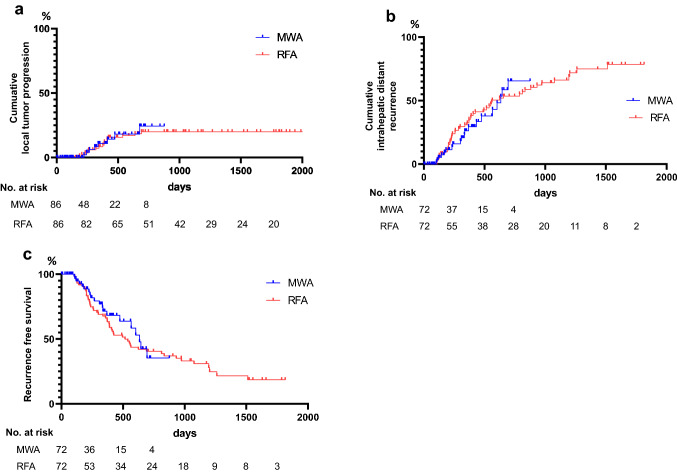
Kaplan–Meier curves of hepatocellular carcinoma patients with microwave ablation (MWA) or radiofrequency ablation (RFA). **a** local tumor progression after matching; **b** intrahepatic distant recurrence after matching; **c** recurrence-free survival after matching

Cumulative LTP rates for 1 and 2 years were 10.9% and 24.4% for MWA, and 8.3% and 19.9% for RFA, respectively (hazard ratio [HR] = 0.87; 95% confidence interval [95% CI] 0.36 to 2.07; *P* = 0.746) (Fig. 1a). Cumulative IDR rates for 1 and 2 years were 26.2% and 65.5% for MWA and 32.4% and 53.5% for RFA, respectively (HR = 1.03; 95% CI 0.61 to 1.73; *P* = 0.890) (Fig. 1b). Cumulative RFS rates for 1 and 2 years were 71.4% and 35.3% for MWA and 66.1% and 40.4% for RFA, respectively (HR = 1.15; 95% CI 0.69 to 1.91; *P* = 0.566) (Fig. 1c). Multivariate analysis, adjusted for propensity scores, showed no significant difference between MWA and RFA in terms of tumor recurrence.

### Complications

One patient each in the MWA and RFA groups had subcapsular hemorrhage after ablation. One liver infarction and one pneumothorax occurred in the MWA group, whereas one cholecystitis and one liver abscess occurred in the RFA group. The complication rate was the same in both groups (4.16% vs. 4.16%). No treatment-related deaths occurred. None of the patients developed local dissemination of the cancer cells along the puncture line.

### Risk factors associated with LTP and RFS

We further investigated the risk factors for LTP and RFS after PTA. The risk of LTP adjacent to large vessels (HR = 3.013; 95% CI 1.46 to 6.186; *P* = 0.002) was the only risk factor associated with PTA (Table 2). In contrast, age, viral hepatitis, recurrent type, ALBI score, AFP, tumor diameter, tumor number, and RFA were identified as risks associated with RFS in univariate analyses. In the multivariate analysis age (HR = 1.873; 95% CI 1.085 to 3.232; *P* = 0.024) recurrent type (HR = 3.329; 95% CI 2.047 to 5.412; *P* < 0.001), ALBI score (HR = 1.633; 95% CI 1.101 to 2.422; *P* = 0.014), and tumor diameter (HR = 2.626; 95% CI 1.175 to 5.867; *P* = 0.018) were identified as independent risks associated with RFS (Table 3).

**Table 2:** Univariate analyses of predictive factors for local tumor progression

Variables	Hazard Ratio (95% CI)	P Value
Background (viral hepatitis)	1.558 (0.641–3.784)	0.328
ALBI (−2.60)	0.801 (0.396–1.622)	0.539
PLT (> 10 × 10^4^)	0.902 (0.45–1.808)	0.771
AFP (> 20 ng/ml)	0.953 (0.441–2.061)	0.904
Tumor size (> 3 cm)	1.133 (0.154–8.314)	0.903
TACE ( +)	0.905 (0.418–1.957)	0.800
Location (vessel-adjacent)	1.866 (1.328–2.621)	0.000
Device (RFA)	0.694 (0.317–1.52)	0.362
Ablation time	1.018 (0.950–1.09)	0.613
Session	1.076 (0.600–1.928)	0.806

*RFA* radiofrequency ablation, *PLT* platelet, *ALBI* albumin bilirubin grade, *AFP* α fetoprotein, *TACE* transcatheter arterial chemoembolization

**Table 3:** Univariate and multivariate analyses of risk factors for cumulative recurrence-free survival

Variables	Hazard ratio (95% CI)	*P* value	Hazard ratio (95% CI)	*P* value
Age (> 65)	1.728 (1.015–2.942)	0.044	1.873 (1.085–3.232)	0.024
Sex (male)	0.981 (0.662–1.454)	0.926		
Background (viral hepatitis)	1.559 (0.988–2.459)	0.056	1.180 (0.719–1.935)	0.511
Naïve (recurrent type)	3.413 (2.121–5.492)	0.000	3.329 (2.047–5.412)	0.000
Child–Pugh (B)	1.09 (0.696–1.706)	0.708		
ALBI (> -2.60)	1.553 (1.069–2.257)	0.021	1.633 (1.101–2.422)	0.015
PT (> 70%)	0.950 (0.627–1.441)	0.811		
ALB (> 3.5 g/dl)	0.813 (0.539–1.227)	0.326		
T-Bil (> 2.0 mg/dl)	0.968 (0.393–2.382)	0.944		
ALT (> 30U/L)	0.918 (0.640–1.316)	0.641		
PLT (> 10 × 10^4^)	0.976 (0.945–1.01)	0.164		
AFP (> 20 ng/ml)	1.442 (0.983–2.116)	0.061		
Tumor size (> 3 cm)	3.818 (1.734–8.41)	0.001	2.626 (1.175–5.867)	0.019
Tumor number	1.149 (0.999–1.321)	0.051	1.130 (0.977–1.308)	0.100
TACE ( +)	0.951 (0.628–1.44)	0.814		
Location (vessel-adjacent)	1.001 (0.657–1.522)	0.998		
Device (RFA)	1.56 (0.963–2.525)	0.070	1.590 (0.956–2.644)	0.074

*RFA* radiofrequency ablation,* PT* prothrombin time,* ALB* albumin,* T-Bil* total bilirubin,* ALT* alanine aminotransferase,* PLT* platelet,* ALBI* albumin bilirubin grade,* AFP* α fetoprotein,* TACE* transcatheter arterial chemoembolization

## Discussion

With the widespread use of surveillance programs for HCC in high-risk patients, patients are increasingly indicated for curative treatment [14]. Heat-based thermal ablations are the best therapies for patients with early-stage HCC who are unsuitable for surgery, although the difference in treatment efficacy between MWA and RFA is unclear, and the choice of therapy is often at the discretion of each institution and operator. Furthermore, with the advent of new MWA systems that eliminate the shortcomings of first-generation systems, the comparison between MWA and RFA is still a meaningful topic. This study applied PSM to reduce the confounding bias of patients’ baseline characteristics when comparing the two therapies. PSM helped illustrate the value of PTA as a treatment for HCC adjacent to large vessels, a major risk factor for local tumor progression. If the target lesion is in the vicinity of a large vessel, PTA cannot sufficiently cauterize the tissue due to the heat sink effect; a phenomenon that occurs when thermal energy diffuses away from the target lesion due to blood flow in adjacent vessels [15]. While the heat sink effect weakens RFA, the faster heating and higher temperature provided by microwave energy allow for a reduced heat sink effect [16, 17]. Huang et al. reported the outcomes of MWA for HCC adjacent to large vessels and found that the risk did not significantly affect LTP and overall survival [18]. Similarly, Potretzke et al. reported that MWA had a lower rate of LTP than RFA [19], and Abdelaziz et al. reported that LTP was significantly lower in MWA than RFA, although overall survival was comparable [20]. On the other hand, Vogl et al. showed no significant differences between the two therapies in terms of complete treatment response, residual foci of untreated disease rate, recurrence rate, or progression-free survival [21].

In this study, before PSM, patients in the RFA group had a worse liver functional reserve, and the proportion of tumors adjacent to large vessels was lower than patients in the MWA group. Therefore, we applied PSM to reduce the confounding bias of these baseline characteristics between the two therapies. After PSM, patients with HCC in both groups were matched in terms of liver functional reserve and tumor location. LTP, IDR, and RFS showed little difference between the two therapies. However, MWA was able to significantly reduce the number of sessions required and ablation time. In addition to being therapeutically beneficial to the patient with less stress, this is also beneficial for the operator. New-generation MWA had been demonstrated to create a large predictable spherical ablation zone in clinical practice [22], and the benefits of spherical ablation may increase the accuracy of ablation strategies. Feng et al. adopted PSM and compared the treatment outcomes of MWA and RFA for perivascular HCC, and reported that RFS was significantly better in the MWA group than in the RFA group, although overall survival was similar [23]. However, the pursuit of local tumor progression after ablation, which is a major concern for perivascular HCC, had not been sufficient. Santambrogio et al. reported that MWA was associated with lower LTP in a retrospective study comparing the efficacy of laparoscopic MWA and RFA for 1–3 HCC tumors smaller than 3 cm in size [24]. However, there may have been a potential bias in the proportion of lesions adjacent/non-adjacent to large vessels in the two treatment groups. In this study, the patient groups’ baseline characteristics were matched, although differences in treatment devices were not identified as risk factors for LTP. Univariate analysis identified the vicinity of large vessels alone, while tumor diameter, combination therapy with TACE, ablation time, and the number of sessions did not pose a risk of local tumor progression. For risk factors associated with RFS, age, recurrent type, ALBI score, and tumor diameter were identified as independent risk factors by multivariate analysis. In this study, patients under the age of 65 years accounted for only 15% of the total study cohort, which may have led to an extraction bias. In other words, it may be the case that younger patients were more likely to choose curative surgery than older patients. Previous HCC therapies, the number of tumors, and the severity of the underlying liver disease were identified as risk factors in HCC recurrence, a finding which is supported by previous reports [25, 26].

Thermal ablation for HCC adjacent to large vessels causes portal vein thrombosis and severe bile duct injury, which can significantly decrease the patient’s quality of life [27]. Given that the new-generation MWA system is less affected by the heat sink effect and allows for a large ablation zone, there were concerns about increased ablation complications for HCC, especially for tumors adjacent to large vessels. However, there was no difference between the two groups in terms of major complications in this study. This observation was similar to previous reports, including a large multicenter study in Italy and a systematic review comparing the complications of MWA and RFA [28, 29].

This study has some limitations. First, the RFA procedure was performed early in the study, while the MWA procedure was performed later, which may have resulted in bias. In addition, it was not possible to compare the long-term treatment outcomes due to the short follow-up period of MWA. Second, we utilized PSM to balance baseline characteristics, although selection bias may have occurred because of the retrospective study design. Third, since this is a single-center study, the generalizability of the results may be limited. However, this preliminary report on the performance of a new-generation MWA system lays the foundation for prospective research regarding the optimal PTA procedure for HCC.

## Conclusion

The therapeutic effect of new-generation MWA was comparable to that of RFA, although MWA has advantages over RFA regarding efficacy, including shorter ablation time and fewer sessions required. Future research is required to accumulate more cases and compare long-term treatment outcomes.

## Data Availability

The datasets used and/or analyzed during the present study are available from the corresponding author on reasonable request.
